# Per- and poly-fluoroalkyl substances (PFAS) effects on lung health: a perspective on the current literature and future recommendations

**DOI:** 10.3389/ftox.2024.1423449

**Published:** 2024-07-18

**Authors:** Megan E. Solan, Jin-Ah Park

**Affiliations:** Department of Environmental Health, Harvard T.H. Chan School of Public Health, Boston, MA, United States

**Keywords:** PFAS, inhalation toxicology, lung disease, PFAS toxicity, airway epithelium

## Abstract

Per- and poly-fluoroalkyl substances (PFAS) are a broad class of synthetic compounds widely used in commercial applications. The persistent nature of PFAS in the environment has earned them the epithet “forever chemicals.” Concerns arise from widespread exposure to PFAS from occupational, household, and environmental sources. This widespread use of PFAS is particularly concerning, as emerging epidemiological evidence highlights their adverse effects on lung health. Such adverse impacts include impaired fetal lung development, reduced immune function in children, and potential links to lung cancer. Both *in vivo* and *in vitro* studies illuminate potential mechanisms underlying such adverse health outcomes subsequent to PFAS inhalation exposure, which may include immunomodulation, oxidative stress, and disruptions to epithelial barriers. However, evidence-based information focusing on the mechanisms of PFAS-mediated lung injury is lacking. Additionally, the discrepancies between data collected from animal and epidemiological studies highlight the need for improved approaches to better understand the toxicity results of PFAS exposure. To address these gaps, we recommend leveraging route-to-route extrapolation for risk assessment, prioritizing research on understudied PFAS, and adopting physiologically relevant, high-throughput approaches. These strategies are aimed at enhancing our understanding of PFAS inhalation effects, aiding in more informed risk management decisions. In this review, we summarize the current literature on PFAS exposure, emphasizing its adverse effects on lung health, particularly through inhalation. We then discuss the current knowledge on mechanisms underlying tissue- and cellular-level adverse outcomes caused by PFAS.

## 1 Introduction

Roy J. Plunkett discovered polytetrafluoroethylene (PTFE) at the Jackson Laboratory of the DuPont Company in 1938 while conducting experiments with chlorofluorocarbon refrigerants (ITRC, 2022). Less than a decade later, synthetic chemistry innovations by the 3M Corporation produced perfluorooctanoic acid (PFOA) in 1947. PTFE and PFOA were the first members of a class of compounds known as per- and poly-fluoroalkyl substances (PFAS), which has grown to include nearly 15,000 members according to the CompTox Chemicals Dashboard (https://comptox.epa.gov/dashboard/chemical-lists/PFASSTRUCTV5). This chemical class has emerged as one of the foremost environmental challenges of the 21st century and is expected to persist for decades. Due to their resistance to environmental degradation and long-term stability, these compounds have been infamously dubbed “forever chemicals” by the mainstream media.

The concern around PFAS in the U.S. was initially driven by findings from an investigation by the Centers for Disease Control (CDC), which found perfluorooctane sulfonate (PFOS) in the serum of 98% of the population (CDC, 2019). PFAS are readily absorbed into the bloodstream and strongly bind to human serum albumin, leading to enhanced distribution throughout the body (Moro et al., 2022). Epidemiological studies indicate that exposure to PFAS leads to numerous adverse health outcomes, including increased cholesterol levels, liver damage, kidney disease, thyroid hormone disruption, immune system alterations, cancers of the kidney and testes, cardiovascular disease, neurological disorders, diabetes, osteoarthritis, and respiratory disease (DeWitt, 2015; ASTR, 2018).

*In vitro* and *in vivo* studies have noted that PFAS exposure results in dysregulation of normal cellular proliferation and apoptosis, reduced lung surfactant function, and impaired lung function (Jabeen et al., 2020; Sorli et al., 2020). Cellular proliferation and apoptosis dysregulation have received greater attention due to their established link to cancer development. Despite the evidence supporting the adverse effect of PFAS on the lung (Sorli et al., 2020), uncertainty remains regarding the impacts of PFAS on respiratory health in humans. This limitation could be partially due to prevailing research primarily focused on legacy PFAS (such as PFOA and PFOS). Moreover, the evidence regarding the adverse effects of exposure is conflicting, highlighting the need for additional studies that establish a link between epidemiological findings and mechanistic investigations (i.e., lab studies evaluating PFAS disposition characteristics such as absorption, distribution, metabolism, and excretion; ADME). To reconcile contradictory evidence, we provide a comprehensive summary that centers on the sources of PFAS inhalation and experimental data related to respiratory health risks. As a result, our review will delineate the existing knowledge gaps in our current understanding of PFAS inhalation toxicity and propose future research directions to comprehensively assess exposure risks.

## 2 Human exposure to PFAS through inhalation

The unique properties of PFAS, which result from chains of polar covalent bonds formed between carbon and fluorine, contribute to their versatile uses in commercial and industrial applications. An exhaustive report by Gaines (2023) summarized the historical and current usage of PFAS, which are found in various household items such as coating products, paints, inks, varnishes, cleaning products, and waxes. PFAS also have a role in ceramic synthesis, adhesives, dry-cleaning systems, electronics manufacturing, and etching processes (Garg et al., 2020). Muensterman et al. (2022) reported that wearing PFAS-treated facemasks for prolonged periods while protecting against COVID-19 may have contributed to human exposure via dermal absorption, inhalation of gas-phase PFAS, and ingestion of particulate-phase PFAS.

**FIGURE 1:**
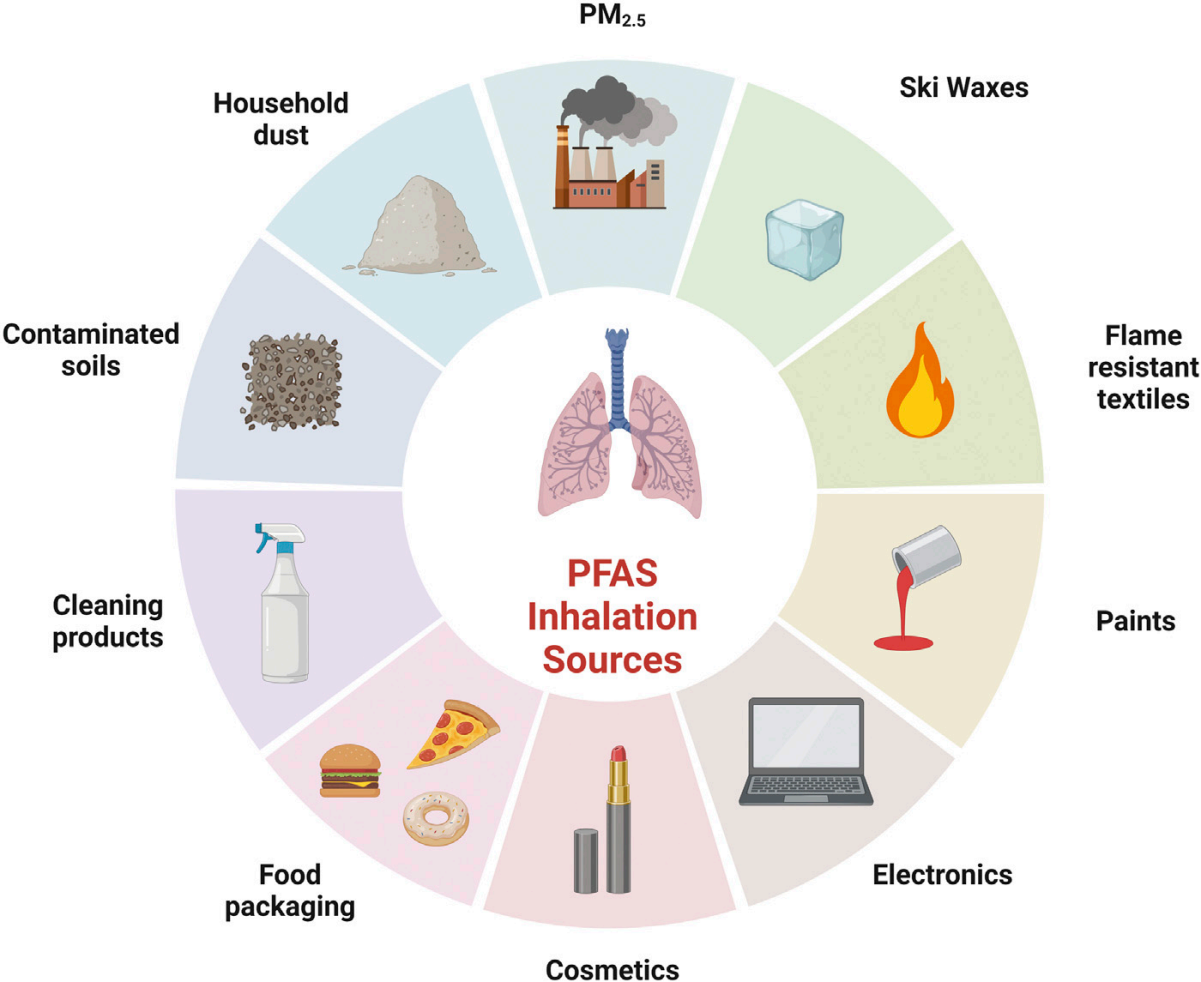
Examples of sources of PFAS inhalation exposure. Created with BioRender.com.

Primary routes of PFAS exposure include consumption of contaminated food and water. However, exposure can also occur through the ingestion of contaminated dust or inhalation of PFAS in the air (ATSDR, 2022). As illustrated in Figure 1, the sources of PFAS exposure are widespread. This section will focus on three primary exposure settings: household, environmental, and occupational. Each exerting a distinctive influence on the nature and degree of PFAS exposure.

### 2.1 Household exposures

In household settings, PFAS are present in a wide range of consumer products, making inhalation exposure unlikely to come from a single-point source. Instead, it results from the combined usage of PFAS-containing products and the generation of particulates due to their wear and tear. As summarized in a review by DeLuca et al. (2022), concentrations of PFAS in house dust vary as follows: 0.76–48.05 ng/g for PFOA, 0.95–62.00 ng/g for PFOS, 0.49–11.85 ng/g for perfluorononanoic acid (PFNA), 0.12–5.55 ng/g for perfluorohexane sulfonic acid (PFHxS), 0.44–8.65 ng/g for PFHxA, 1.37–13.90 ng/g for PFBA, and 0.44–0.81 ng/g for PFBS. Until now, only two studies in the meta-analysis reported the data with indoor air concentrations. Among the PFAS chemicals, PFOA exhibits the highest median concentrations, ranging from 0.02 to 0.05 ng/m^3^ (Makey et al., 2017; Balk et al., 2019). Besides PFOA, other PFAS concentrations vary from 0.0024 ng/m^3^ to 0.0015 ng/m^3^ for PFNA, with a median concentration of 0.0012 ng/m^3^ for PFOS and 0.01 ng/m^3^ for perfluorohexanoic acid (PFHxA).

Although information on the accessible fraction of PFAS in dust particles is limited, the associations between household dust concentrations and serum measurements are available. A meta-analysis by Norgaard et al. (2014) revealed that median concentrations of study populations from the U.S. and Canada range as follows: 1.01–4.50 ng/mL for PFOA, 1.55–11.60 ng/mL for PFOS, 0.36–2.21 ng/mL for PFNA, and 0.22–1.70 ng/mL for PFHxS. The review from DeLuca et al. (2022) concluded that contaminated household dust could account for 13%, 3%, 7%, and 25% of PFAS serum concentrations of PFOA, PFOS, PFNA, and PFHxS, respectively. Fraser et al. (2013) assessed serum PFHxA and perfluorobutanesulfonic acid (PFBS), reporting concentrations of 0.03 ng/mL and 0.04 ng/mL, respectively. Overall, PFHxS represented a notable serum level attribution; in adults, the percentage of PFHxS serum concentrations attributable to contaminated household dust ranges from 0.73% to 41.92%, while in children, it ranges from 39.92% to 54.76% (Balk et al., 2019; Poothong et al., 2020). Another review highlights considerably higher serum concentrations of PFAS resulting from contaminated house dust in children compared to adults, indicating the significance of incidental ingestion of indoor dust for young children (Juhasz et al., 2023). However, a lack of comprehensive analysis on multiple types of PFAS and the accessible fraction from dust poses a challenge to designing studies to assess the effects of PFAS from contaminated household media.

### 2.2 Occupational exposures

Occupational exposure to PFAS occurs during the production and manufacturing of PFAS-containing items. The broad applications of PFAS pose potential risks to individuals across various sectors and industries, including consumer product manufacturing, construction, mining, and military applications (Gaines, 2023). In particular, occupational exposure among fluorochemical plant workers has been extensively studied, with the primary route of exposure believed to be via dermal absorption; however, inhalation can occur from aerosolization of PFAS-containing products (Lucas et al., 2023). Nilsson et al. (2010) demonstrated that inhaling airborne particles and fumes containing a blend of gaseous fluoropolymers can contribute to PFAS blood levels in occupational scenarios. It has been speculated that heating PFAS-containing products may further compound inhalation risks associated with exposure by increasing the volatility (Mazumder et al., 2023; Crawford and Hartmann, 2024).

A review by Paris-Davila et al. (2023) explored exposure to PFAS in various occupational settings, including ski-waxing cabins, textile manufacturing plants, fire stations, office buildings, and custodial activities. The review included data on PFAS levels in air and dust, revealing significant variations across different workplaces. When compared to all other reported occupational and residential exposures, ski waxing cabins showed the highest overall PFAS air concentrations, with levels of PFOA, PFHxA, perfluorododecanoic acid (PFDoDA), and perfluorodecanoic acid (PFDA) detected as high as 1 μg/m^3.^ In addition, dust samples from these cabins showed concentrations of PFAS as high as 10 mg/g (Freberg et al., 2010; Nilsson et al., 2010). Ski waxing involves melting wax onto skis with a hot iron (>120°C), followed by scraping off excess wax, thereby releasing volatile PFAS into the air, posing health risks through dermal absorption and inhalation (Crawford and Hartmann, 2024).

Assessments of PFAS particulates in different occupational settings showed varying air concentrations. For instance, floor waxing during custodial activities produced the lowest air concentrations compared to other studies, often falling below detection limits (Zhou et al., 2022). In textile manufacturing settings, PFAS concentrations in air were consistently between 0.01 and 0.0001 μg/m^3,^ with exceptionally high levels of PFTetDA levels observed during specific processes, including scouring, drying, and heat setting, reaching concentrations of 10 μg/m^3^ (Heydebreck et al., 2016; Wu N. et al., 2019). Another significant area of occupational exposure assessments focuses on firefighters who are exposed to PFAS through the use of firefighting foams, such as aqueous film-forming foam (AFFF). Paris-Davila et al. (2023) observed that the concentrations of PFAS in air and dust samples collected from fire stations are similar to those detected in residential areas, as reported by Wu N. et al. (2019). Despite comparable concentrations of PFAS detected in fire stations and residential areas, the median levels of PFOS and PFHxS in the serum of firefighters are generally higher than those reported by the National Health and Nutrition Examination Survey (NHANES) population data (Burgess et al., 2023; Lucas et al., 2023). This discrepancy indicates that the exposure of firefighters to PFAS likely occurs during firefighting activities rather than from the air or dust in the fire stations. PFAS used in firefighters’ gear and exposure to smoke presents significant inhalation risks. Heating of PFAS-containing products and combustion of consumer products at the scene of a fire can increase these risks by increasing volatility (Nilsson et al., 2010; Peaslee et al., 2020; Mazumder et al., 2023; Crawford and Hartmann, 2024).

Given the importance of occupational exposure, further research in a broader range of occupational settings is necessary to develop informed recommendations for exposure mitigation measures. However, precise assessments of inhalation exposure levels in firefighters, workplaces using fluoropolymer waxes, and textile manufacturing professions, where exposure to PFAS is known to be relatively high, are crucial for comprehensive risk assessment and management. Considering the extreme conditions that firefighters are subject to, identifying the level of PFAS inhalation exposure from the soot and smoke of commercial products should be explored further.

### 2.3 Environmental exposures

Environmental contamination of drinking water supplies by PFAS is particularly problematic due to their long half-life in the body. For example, the estimated median elimination half-lives of PFOS, PFOA, PFNA, and PFHxS range from 2.1 to 8.5 years (Hu et al., 2016). However, initiatives targeting local drinking water clean-up and the phase-out of legacy PFAS (such as PFOA and PFOS) have yielded positive outcomes. Over the past two decades, these mitigation efforts have led to declining trends of PFAS in serum concentrations among highly exposed populations (Miaz et al., 2020). However, alongside the reduction of legacy PFAS in serum and drinking water, a rise in unidentifiable extractable organic fluorine (EOF) has been observed (Hu et al., 2019). The increase of EOF emphasizes the imperative for further research to explore the interplay between non-target PFAS, their precursors in various matrices beyond drinking water, such as outdoor air, and their implications for biological exposures.

PFAS concentrations in outdoor air exhibit a wide range from undetectable to hundreds of picograms per cubic meter (pg/m^3^) (Shoeib et al., 2005; Jahnke et al., 2007; Rauert et al., 2018; De Silva et al., 2021). Despite the relatively low concentrations detected, outdoor air exposure still warrants some scrutiny due to the higher mobility of PFAS in air relative to soil and groundwater (De Silva et al., 2021). In studies using sophisticated models to estimate PFOA concentrations in ambient air and water in the communities around a heavily contaminated area in West Virginia, PFAS levels in indoor air were only 10% of those in outdoor air because of partial infiltration (Shin et al., 2011a; Shin et al., 2011b). The results indicated that exposure to PFAS through inhalation might exceed it through water ingestion in areas with continuing air emissions. Fluorotelomer alcohols (FTOHs) are the most frequently studied PFAS in outdoor air due to their high volatility and often dominance in air samples (Shen et al., 2023).

Additionally, as regulations evolve, novel PFAS are identified in particulate matter (PM_2.5_). For example, hexafluoropropylene oxide-dimer acid (HFPO-DA, also known as GenX) has been detected in air sampling studies at concentrations up to 21.5 pg/m^3^ (Lin et al., 2020; Faust, 2023). Despite the emergence of new PFAS, PFOA and PFOS remain the dominant species in outdoor air samples, with levels as high as 14.1 pg/m^3^ in ambient air collected in North Carolina in 2019—nearly two decades after manufacturing of legacy PFAS in the region stopped (Zhou et al., 2021).

Moreover, Pan et al. (2023a) reviewed literature from studies assessing the concentrations of PFAS in PM_2.5_ collected from indoor and outdoor air. Their report revealed that PFAS is a significant constituent of the organic compounds detected in PM_2.5_, with a dramatic increase in the detection of newer PFAS, such as HFPO-DA. The review also noted that PM_2.5_ from urban areas often contains higher PFAS concentrations than that from rural areas. Furthermore, the review highlighted the significant role of PM_2.5_ in PFAS exposure via inhalation and the need to consider the possible synergistic adverse effects on lung health.

## 3 Effects of PFAS on lung health

### 3.1 Fetal lung development

During embryonic development, the functional growth of the lungs relies on the synchronized progression of vital processes such as cellular differentiation and proliferation, branching morphogenesis, alveolarization, and the maturation of pulmonary, immune, vascular, and neural systems (Kajekar, 2007). Consequently, exposure to environmental pollutants during pivotal prenatal and postnatal development stages can significantly influence the trajectory of lung morphogenesis and maturation (Zepp and Morrisey, 2019). In humans, PFAS tends to accumulate at the highest concentrations in fetal lung tissues, raising particular concerns regarding potential associations between prenatal PFAS exposure and children’s respiratory health (Mamsen et al., 2019). As lung development is an ongoing process that commences in the early stages of embryonic life and extends through adolescence, any factors that disrupt the developmental program at any stage may lead to changes in lung function and an increased risk of disease later in life (Kajekar, 2007).

According to *in vivo* studies, fetal lung tissue may be a sensitive target of PFAS-mediated toxicity during perinatal exposure (Borg et al., 2010). For example, PFOA exposure hinders lung development in offspring by inducing changes in genes associated with the cytoskeleton, extracellular matrix, lipid metabolism, and secreted proteins in the rat fetal lung (Borg et al., 2010; Chen et al., 2012; Ye et al., 2012). Moreover, it is possible that gestational exposure to PFOS induces lung defects potentially through the upregulation of nucleotide-binding oligomerization (NOD-) like receptors receptor protein 3 (NLRP3) inflammatory factor or downregulation of hypoxia-inducing factor 1α (HIF-1α) and vascular endothelial growth factor A (VEGF-A) (Zhang H. et al., 2021). During embryonic development, HIF-1α and VEGF-A are both highly expressed in epithelial cells of bronchi and alveolar ducts. Thus, the downregulation of these factors would result in reduced pulmonary capillary and alveolar development.

While animal studies provide compelling evidence of the adverse effects of PFAS exposure on fetal development, the epidemiological evidence lacks consensus regarding these impacts on developing children. Philippat et al. (2023) analyzed up to 13 PFAS as mixtures and individually in maternal serum collected during pregnancy to determine the associations between prenatal exposure to PFAS and respiratory health in 2 and 3-year-old children but found no association. Similarly, Atagi et al. (2023) found no clear associations between prenatal PFAS exposure and the prevalence of wheezing and asthma in 4-year-old children.

### 3.2 Lung function in children and adults

In children, PFAS can induce immunosuppressive effects, potentially affecting their antibody responses to vaccinations, especially with exposures to PFOA, PFOS, and PFHxS. Kvalem et al. (2020) identified sex as a potential factor in some exposure-health associations, reporting that perfluoroheptanoic acid (PFHpA), PFOA, perfluoroheptanesulfonic acid (PFHpS), and PFOS showed positive correlations with infections of the lower respiratory tract, but the risk of infection demonstrated a stronger relationship with PFOA in girls and for PFHpS and PFOS in boys. Furthermore, PFNA and PFHpS exhibited strong positive associations with rhinitis in girls. Despite these findings, the biological mechanisms underlying these sex differences remain unclear.

Elevated serum PFAS levels are also linked to dysregulation of T-helper (TH) cells, impacting the secretion of type 1 and type 2 cytokines that are critical for asthma development (Zhu et al., 2016). Notably, a cross-sectional study (Jackson-Browne et al. (2020) revealed a weak association between serum PFAS concentrations and increased asthma prevalence in US children between 3 and 11 years of age. They also found that age serves as a factor in modifying the associations between serum PFOA and PFOS concentrations and asthma. In a murine model Ryu et al. (2014), found that inhalation exposures to PFOA induces airway hyperresponsiveness (AHR) and increases lung macrophages. PFOA and PFOS, however, do not affect ovalbumin (OVA)-induced AHR, suggesting that PFAS exposure is not a risk factor for developing more severe allergic asthma-like symptoms. Dermal exposures to PFOA, however, have been observed to enhance the hypersensitivity response to OVA suggesting that PFOA exposure increases sensitivity to environmental allergens (Fairley et al., 2007). Despite some exceptions, most studies failed to establish a clear association between PFAS exposure and asthma (Authority, 2020; Schrenk et al., 2020). In some cases, contradictory findings have been reported in studies assessing the impact of childhood PFAS exposure on asthma and allergies (Dong et al., 2013; Averina et al., 2019; Jackson-Browne et al., 2020; Kvalem et al., 2020; Pan Z. et al., 2023).

In comparison to studies in children, there are far fewer studies on adverse outcomes associated with adult PFAS exposure and lung function. Case reports have demonstrated that inhaling PFAS-impregnated sprays causes acute toxicity in humans, resulting in immediate symptoms such as coughing, difficulty breathing, chest discomfort, and decreased blood oxygen levels (Scheepers et al., 2017). However, a cross-sectional study using the 2011–2012 U.S. population PFAS serum from NHANES datasets found no association between PFAS exposure and a decline in lung function (Heinle et al., 2019).

### 3.3 Lung cancer

In terms of PFAS exposures and their health impacts, one notable concern is cancer. The International Agency for Research on Cancer (IARC) recently classified PFOA as “carcinogenic to humans (Group 1),” citing “strong” mechanistic evidence in exposed humans, including epigenetic alterations and immunosuppression (Zahm et al., 2024). Additionally, the Environmental Protection Agency (EPA) has assigned carcinogenic potential classifications to PFOA, PFOS, and a novel alternative, HFPO-DA (GenX) (USEPA, 2016; 2021). Occupational exposure studies have revealed that lung cancer accounts for the highest number of deaths among specific cancer types reported in PFAS occupational mortality studies (Steenland and Woskie, 2012; Steenland and Winquist, 2021). Furthermore, PFOA-contaminated water districts exhibit a 20%–30% higher risk of lung cancer and significantly elevated site-specific cancer odds ratios compared to non-contaminated water districts in the same regions (Vieira et al., 2013).

Recently, Moon and Mun (2024) conducted an analysis using NHANES data to explore the potential links between exposures to PFAS compounds—specifically PFOA, PFOS, PFHxS, and PFNA—and various types of cancer. The study employed logistic regression and directed acyclic graph adjustments to assess serum concentrations of PFAS compounds in relation to cancer diagnoses, including lung cancer. The results revealed statistically significant odds ratios for ln (PFOS) and ln (PFNA) concerning lung cancer, with O.R.s of 2.62 (95% CI 1.24–5.83) and 2.38 (95% CI 1.00–5.52), respectively. These findings provide further evidence for a potential link between these PFAS compounds and an increased risk of lung cancer. However, it is essential to note that O.R.s alone do not establish causation but indicate associations that warrant further mechanistic studies.

While evidence supports that PFAS exposure elevates the risk of lung cancer, the precise pro-carcinogenic effects on lung tissues and cancer risks remain unknown in general (Boyd et al., 2022). It is worth noting that the existing studies have generally focused on a handful of model PFAS, including PFOA, PFOS, PFHxS, and PFNA (Humblet et al., 2014; Jackson-Browne et al., 2020; Luo et al., 2020). Future studies that involve a broader range of PFAS will be essential to adequately elucidate the lines of evidence regarding the development and pathogenesis of lung diseases, including asthma and lung cancer.

## 4 Lung-specific effects of PFAS at cellular- and tissue-levels

### 4.1 Epithelial barrier function

Following inhalation of pollutants, the airway epithelium and lung surfactant act as crucial physical barriers to prevent the entry of harmful substances into the bloodstream. In particular, lung surfactants maintain surface tension to protect against the collapse of the lung at the air-liquid interface in the alveoli during respiration (Zuo et al., 2008; Perez-Gil and Weaver, 2010). Interestingly, PFAS are known for their ability to reduce surface tension efficiently, a property exploited in various industrial applications (Dong et al., 2021). However, this ability of PFAS alludes to potential disruptions to the surface-active films of natural lung surfactants in the alveoli (Sorli et al., 2020).

Evidence for PFAS exposure affecting epithelial barrier function has been observed *in vivo* and *in vitro* in the airway. For example, in mice, perfluoroisobutene (PFIB) increases the permeability of the blood-air barrier by decreasing levels of tight junction proteins between the epithelial cells (Meng et al., 2011). In human bronchial epithelial cells (16HBE), PFOS disrupts epithelial integrity as early as 4 h, as indicated by decreased transepithelial electrical resistance (TEER) and increased dextran-FITC permeability (Lucas et al., 2022). Additionally, PFOS exposure reduces the expression and organization of tight junction proteins, occludin and zonula occludens 1 (ZO-1), which collectively results in a breakdown of the epithelial barrier and an increase in permeability (Li et al., 2012; Zhang Y. et al., 2021; He et al., 2021). In BEAS-2B cells, PFOA alters membrane potential and activates the NLRP3 inflammasome (Dragon et al., 2023). The collective evidence provides valuable insights into how PFAS compromise the integrity of the airway epithelium, indicating a risk of airway damage in highly exposed individuals.

### 4.2 Immunomodulation

Although few studies have explored the mechanisms by which PFAS modulate pulmonary immunity, it has been suggested that PFAS may modulate innate immunity and inhibit acquired immunity (Qazi et al., 2009). *In vitro* studies with human bronchial epithelial cells (BEAS-2B) reveal that PFAS mixtures containing PFOA, PFOS, PFBA, PFBS, and GenX increase the secretion of caspase-1 and HMGB1 and steady-state mRNA expressions of NLRP3, IL-6, IL-5, and IL-8 (Dragon et al., 2023). *In vivo* studies with mice demonstrate that 21-day oral exposures of PFOA in drinking water decrease CD8^+^ lymphocytes while increasing CD4^+^ lymphocytes (Son et al., 2009).

Similarly, dietary exposures to PFOS in mice activate AIM2, releasing mtDNA via the Ca^2+^-PKC-NF-κB/JNK-BAX/BAK axis, leading to IL-1β secretion and tissue damage (Wang et al., 2021). In mice, daily gavage administration of PFOS for 60 days decreases type 1 cytokines, indicating the cause of an imbalance in immune response (Dong et al., 2011). In male mice, GenX exposure leads to increased mitosis in macrophages in bronchoalveolar lavage fluid (BALF) and alveolar epithelial cells (Lee et al., 2022). Increased mitosis in lung cells links to fibroproliferative diseases like fibrosis and increases susceptibility to lung diseases (Morse et al., 2019). Thus, GenX-induced mitosis suggests that PFAS may have immunomodulatory effects on innate and adaptive immune responses. Studies *in vitro* and *in vivo* highlight the potential health risks associated with PFAS exposure, particularly its impact on lung immune function and, subsequently, on the development of lung disease.

### 4.3 Oxidative stress

Persistent inhalation of toxic substances produces reactive oxygen species (ROS) by epithelial cells as a result of an imbalance between cellular oxidants and antioxidants (Albano et al., 2022). An excessive generation of ROS promotes inflammatory processes and lung tissue, contributing to tissue remodeling. Although *in vitro* evidence regarding oxidative stress in lung cells is insufficient, the potential for oxidative damage resulting from PFAS exposure has been reported in other cell types. Using human cell lines derived from the kidney (HEK293-hTLR2), liver (HepaRG), microglia (HMC-3), and muscle (RMS-13), Solan et al. (2023) demonstrated cellular responses to PFBS, PFHxA, PFHxS, HFPO-DA, and 6:2 FTOH. In the liver and muscle cell lines, PFBS exposure increases glutathione peroxidase activity. Notably, all of the PFAS tested induces superoxide dismutase (SOD) enzyme activity. SOD enzymes function as the first line of defense against oxygen-derived free radicals and inhibit oxidative damage in mitochondria (Demirci-Cekic et al., 2022). Thus, the findings from Solan et al. (2023) imply the plausibility of PFAS exposure exacerbating certain lung diseases through the compromised antioxidant mechanisms. Investigating the impact of PFAS on ROS generation and antioxidant enzymes is a necessary next step to understanding the link of PFAS to oxidative stress-related effects on cellular dysfunction.

## 5 Discussion

The conflicting evidence surrounding the adverse effects of PFAS exposure underscores the necessity for further studies that can bridge the gap between epidemiological findings and mechanistic investigations. This comprehensive review provides an assessment of the sources of PFAS inhalation exposure and an in-depth summary of experimental data indicating the potential adverse respiratory outcomes. In this final section, we recommend the area of future research focuses: 1) the implementation of route-to-route extrapolation method, 2) the expansion of knowledge on lesser-studied PFAS, and 3) the promotion of high throughput approaches that are more physiologically relevant. These recommendations are broad suggestions for advancing our understanding of the negative impact of PFAS inhalation, as elaborated below.

### 5.1 Route-to-route extrapolation

The paucity of information on PFAS exposure via inhalation has limited the development of guidelines and acceptable exposure limits that are consistent with risk-based management decisions. Route-to-route extrapolation using oral exposure information is one approach that risk assessors employ when route-specific toxicity data are absent for a particular compound (USEPA, 2009). Utilizing currently available oral toxicity and toxicokinetic data for PFOA and PFOS (Monnot et al., 2023), argue that route-to-route extrapolation may be suitable to derive inhalation-based standards. Based on their analysis, the authors suggested that an air concentration of 0.07 μg/m^3^ would be a sufficient reference concentration. They posit that values determined for longer chain PFAS might potentially provide protect for shorter chain PFAS.

### 5.2 Broadened PFAS research

PFAS are diverse, with many structures lending to different properties, which would ultimately impact their toxicokinetics and disposition following exposure. Only a handful of PFAS have adequately characterized toxicokinetic data (Sanchez Garcia et al., 2018; Wu Y. et al., 2019; Patlewicz et al., 2019; Pizzurro et al., 2019)– a component critical for quantitative risk assessments. While legacy PFAS are still relevant in environmental exposures, more attention should be given to quantifying exposure to less-studied PFAS, including short-chain and alternative PFAS. Advancements in human toxicokinetic studies and more data on the lesser-studied PFAS are the next critical step to establish the exposure limit that protects public health.

### 5.3 Cell-based and high-throughput approaches

In assessing the potential for adverse outcomes associated with PFAS inhalation exposure, it is essential to recognize that the existing body of evidence predominantly relies on animal studies, which may not fully capture the actual risk to human health. Relying on animal models to predict the effects of inhalation exposure is especially challenging due to significant physiological, anatomical, and metabolic differences between species, such as variations in airway structures, breathing patterns, cell types and compositions, and metabolic capacities. For example, the inherent differences in PFAS elimination and half-lives between humans and rodents require conducting rodent studies at concentrations that levels far exceeding realistic human exposures (Pizzurro et al., 2019). Thus, animal studies may not align with the findings from epidemiological studies. Mechanistic *in vitro* studies, however, have demonstrated promise in human health risk assessment and offer a potential solution to the challenges posed by the substantial variability in toxicokinetics and toxicity across different species. Moreover, *in vitro*, high-throughput screening (HTS) methods offer the potential to generate reliable data from toxicity screening on a greater number of chemicals, while enhancing replicability and cost-effectiveness (Solan and Lavado, 2022). To take advantage of HTS, the approaches should seek to encapsulate the complex pulmonary physiology in culture systems. Utilizing 3D culture systems (e.g., spheroids) and co-culture models can advance our understanding of complex interactions between different cell- and tissue types. Multi-organ chips are anticipated to address the limitations of conventional *in vitro* models, facilitating research on the impact of air pollution on the body and the early stages of drug development (Zink et al., 2020; Jalili-Firoozinezhad et al., 2021). These devices emulate the physiological structure of internal organs and their interactions with soluble metabolites, allowing for *in vitro* simulation of interactive effects between organs.

For *in vitro* models accurately predict PFAS toxicity in a regulatory context they must be thoroughly validated and standardized by regulatory agencies. Rapid accumulation of robust data on short-chain PFAS alternatives is crucial for informed decision-making and safety assessments. Learning from the delayed recognition of the harmful effects of legacy PFAS, it is imperative to advance our knowledge regarding the physicochemical properties of short-chain PFAS to ensure the safety of public health.
